# Evaluation of Changes in Cardiac Troponin I Levels After Direct Current Cardioversion in Patients With Atrial Fibrillation

**DOI:** 10.7759/cureus.67162

**Published:** 2024-08-18

**Authors:** Marina Katerini, Christine Politi, Olympia Konstantakopoulou, Eleni Kyritsi, Evgenia Minasidou, Lambrini Kourkouta, Konstantinos Koukourikos, Areti Tsaloglidou

**Affiliations:** 1 Cardiology Clinic, General Hospital of Pella (Hospital Unit of Edessa), Thessaloniki, GRC; 2 Nursing, National and Kapodistrian University of Athens, Athens, GRC; 3 Cardiology Clinic, Hippokration General Hospital, School of Medicine, National and Kapodistrian University of Athens, Athens, GRC; 4 Nursing, International Hellenic University of Thessaloniki, Thessaloniki, GRC

**Keywords:** troponin i level, myocardial injury, cardiac troponin i, atrial fibrillation, synchronised direct current cardioversion

## Abstract

Introduction: Direct current cardioversion (DCCV) of atrial fibrillation (AF) is a procedure used to restore normal heart rhythm. Cardiac biomarkers, such as cardiac troponin I (cTnI), are elevated in situations where injury-myocardial cell necrosis is induced.

Aim: The aim of the present study was to investigate the change in cTnI levels, i.e., whether a myocardial injury is present, in patients with AF whose elective treatment was synchronized DCCV via a biphasic defibrillator.

Methods: The study sample included 59 patients who underwent synchronized DCCV for AF reversion. Measurement of cTnI before and after DCCV (one, three, and six hours) was performed by blood sampling and subsequent assay.

Results: It was observed that the value of cTnI did not change (<0.1 ng/mL) after DCCV at the measurement time points (one, three, and six hours). In addition, the value of cTnI remained constant (<0.1 ng/mL) in relation to the energy delivered, before DCCV and after DCCV (one, three, and six hours). However, it was found that there was a correlation between the outcome (AF reversion or not) and the energy used (joules).

Conclusions: Synchronized DCCV with a biphasic defibrillator did not cause myocardial injury in any of the patients, as there was no change in cTnI values before and after DCCV.

## Introduction

Atrial fibrillation (AF) is the most common type of arrhythmia. Despite advances in the management of patients with AF, it remains one of the leading causes of vascular stroke, heart failure (HF), sudden death, and cardiovascular morbidity worldwide. The incidence and prevalence of AF are increasing worldwide due to an aging population and increased survival of people with chronic diseases. Its high prevalence represents an enormous economic and social burden worldwide [1]. In 2010, the estimated number of men and women with AF worldwide was 20.9 million and 12.6 million, respectively [2]. AF affects 37.574 million people worldwide and its incidence has increased by 33% in the last 20 years. It is estimated to affect six to 12 million people in the Americas by 2050 and 17.9 million in Europe by 2060 [3].

The European Society of Cardiology has classified AF into five types, based on the onset, duration, and cessation of episodes, as follows: (1) newly diagnosed AF is when the arrhythmia has not been previously diagnosed, regardless of its duration or the onset/severity of associated symptoms; (2) paroxysmal AF is when the arrhythmia stops spontaneously or with intervention within seven days; (3) persistent AF is defined as AF that persists for more than seven days, including episodes terminated by either medical or electrical cardioversion (ECV) after seven days; (4) long-term persistent AF is defined as AF that persists for more than 12 months and for which a decision has been made to pursue a rate control strategy; and (5) permanent or chronic AF is defined as AF in which a decision has been made (with the patient's consent) not to attempt to restore sinus rhythm, regardless of its duration. If the decision to resuscitate is changed, the AF should be reclassified as long-term persistent AF [4].

Cardioversion is part of the management of AF and atrial flutter (AFL) in symptomatic patients following a rhythm control strategy and is achieved either by a synchronized direct current (DC) electrical shock (ECV) or pharmacologically with antiarrhythmic medication [5]. Both methods are used worldwide to terminate AF and restore normal sinus rhythm [6]. External ECV terminates episodes of AF in over 90% of cases and is considered the treatment of choice for patients with severe hemodynamic instability and new-onset AF. ECV is performed with short intravenous sedation with midazolam or propofol, and the patient is connected to a monitor for continuous monitoring of blood pressure and oxygen saturation during the procedure [7]. ECV with a biphasic defibrillator has better results, as does pre-treatment with anti-arrhythmic drugs prior to ECV [8,9]. After a successful ECV and as long as the rhythm is monitored for at least three hours, the patient can safely return home. However, there are some cases where it is necessary to monitor the patient for a few hours due to certain minor complications that may occur after ECV such as atrial and ventricular premature beats and muscle soreness [10]. There are also other complications related to sedation, hypotension, the occurrence of ventricular fibrillation due to inappropriate synchronized shock, episodes of bradycardia (usually diagnostic such as sinus node disease or atrioventricular node disease), and tachycardia such as AFL with 1:1 atrioventricular (AV) conduction or torsade de pointes arrhythmias [7].

It should be noted that anticoagulation is necessary before cardioversion to prevent stroke [11]. Pharmacological cardioversion converts recent onset or paroxysmal AF within a few hours in 50-70% of cases. This is achieved by giving patients sodium channel blockers (mainly propafenone or flecainide) or vernakalant. These drugs are not effective in longer-lasting AF [12].

Proteins are part of the cardiac biomarkers that are released into the blood when the heart muscle is damaged. They are essential for the diagnosis, risk assessment, and treatment of patients with chest pain, acute coronary syndrome, or relapse of acute HF. The biomarker used to detect myocardial injury is troponin. Troponin is a complex made up of three components: troponin C (TnC), the calcium-binding subunit; troponin I (TnI), which stops muscle contraction in the absence of calcium; and troponin T (TnT), which binds the troponin complex to tropomyosin. Cardiac troponin I (cTnI) is released into the blood after myocardial necrosis or injury and is therefore detected in the blood by a specific and sensitive method [13]. Myocardial isoform cTnI levels increase within four to six hours after myocardial infarction and then decrease to normal levels within five to seven days [14]. In addition to the diagnostic and prognostic role of cardiac troponin biomarkers in the occurrence of acute myocardial infarction (AMI), elevated cardiac troponin levels may occur in cases of non-ischemic myocardial injury, including sepsis/systemic inflammatory responses syndrome (SIRS) and critical illness, pulmonary embolism, end-stage renal disease, rhabdomyolysis, exercise, burns, drug toxicity, and stroke [15].

Aim

The aim of the present study was to determine whether direct current cardioversion (DCCV) causes a significant increase in cTnI levels indicative of myocardial injury.

## Materials and methods

Study design

This study is a prospective study conducted in the Cardiology Clinic of Edessa General Hospital. The study was designed by the nurse researchers in collaboration with the Director of the Cardiology Clinic and was based on the protocol followed by the Cardiology Clinic for patients undergoing DCCV, which is described below.

Patients admitted to the hospital with AF of unknown onset undergo scheduled DCCV after treatment with anticoagulants at appropriate doses for at least three weeks [16]. They are then admitted to the coronary care unit of Cardiology Clinic and pharmacological cardioversion is attempted. If this is unsuccessful, they will undergo DCCV the following day. For episodes of persistent AF, DCCV is performed within 48 hours of symptom onset if pharmacological cardioversion has failed. DCCV is performed on the same day of admission after a transesophageal echocardiogram to exclude the presence of intra-abdominal thrombi [17].

All cardioversions are performed in the coronary care unit in the presence of an anesthetist, cardiologist, and nurse. The preparation procedure involves connecting the patient to a monitor and electrocardiograph, administering oxygen, and then sedating the patient with intravenous anesthetic (propofol). Immediately afterwards, the cardiologist performs a synchronized DCCV using a biphasic defibrillator, and the patient is awake a few minutes after the DCCV is complete. As there is no optimal energy protocol for achieving sinus rhythm in DCCV of AF [18], patients typically receive a maximum of five DCCV attempts with successive increases in the cumulative energy delivered (50, 100, 150, 200, and 360 joules).

Sample

The study population included 59 men and women over 18 years old who were admitted to the Cardiology Clinic of Edessa General Hospital with AF and underwent synchronized DCCV between May 30, 2022, and July 30, 2023.

Collection of data and measures

Demographic and clinical characteristics were collected from the patient's medical records. The data were collected from the electronic medical records of inpatients in the hospital. Previous medical history, baseline clinical characteristics, initial diagnosis, and follow-up clinical results were included in the database. The data were collected and processed by two nurse researchers in the clinic by taking a blood sample and analyzing it by cTnI test before DCCV and one hour, three hours, and six hours after DCCV.

Inclusion criteria

The patients included in the study were those over 18 years old, patients willing and able to participate in the study, patients admitted with confirmed AF (newly diagnosed, paroxysmal, persistent, or long-term persistent) or after referral by a private cardiologist or primary care cardiologist, patients undergoing planned DCCV, and patients admitted with recent AF (≤48 hours) and after unsuccessful pharmacological cardioversion. In the latter case, a transesophageal echocardiogram was performed.

Exclusion criteria

The patients excluded in the study were those with kidney disease (≥2.5 mg/ml), patients with heart failure, patients with cancer, patients with debilitating mental and psychiatric conditions, hemodynamically unstable patients due to supraventricular arrhythmia, and patients with chronic AF.

Ethical and moral issues

The study protocol was reviewed and approved by the Scientific Council of the General Hospital of Edessa (approval number: 4661/13.5.2022). Written informed consent was obtained from all participants prior to their inclusion in the study. The study was conducted with respect for the patients and the confidentiality of the data collected, as guaranteed by Regulation (EU) 2016/679 of the European Union on the protection of natural persons with regard to the processing of personal data and the free movement of such data.

Statistical analysis

Categorical variables are presented as absolute (n) and relative (%) frequencies, while quantitative variables are presented as means (standard deviation). The Kolmogorov-Smirnov test and normality plots were used to test the normal distribution of quantitative variables. The x2 test (chi-squared test) or Fisher's exact test was used to investigate the existence of a relationship between two categorical variables. To investigate the existence of a relationship between a quantitative variable and a dichotomous variable, the Student's t-test was used when the quantitative variable followed a normal distribution, and the Mann-Whitney test was used when the quantitative variable did not follow a normal distribution. Data analysis was performed using SPSS version 18.0 statistical software (SPSS Inc, Chicago, IL).

## Results

Descriptive results

The study population consisted of 59 patients who developed AF, the majority of whom were males (72.9%, 43 patients). All patients either presented to the emergency department with palpitations and were subsequently hospitalized for a recorded episode of AF in the Cardiology Clinic or were scheduled to undergo DCCV. The mean age of the patients was 66.3 years (SD = 13.0) and a family history was reported by 28.8% of the patients. Of the patients, 28.8% were active smokers, 62.7% were non-smokers, and 8.5% had quit smoking. Of the patients, 78% had arterial hypertension, 39% had diabetes mellitus, 44.1% had dyslipidemia, and 13.6% had thyroid disease. Coronary artery disease was present in 22% of the sample, 5.1% had chronic renal failure, and 8.5% had valvular heart disease. Regarding the type of AF, 25.4% of the sample had first-time (first-diagnosed) AF, 67.8% had persistent AF, and 6.8% had long-standing persistent AF. A total of 94.9% had successful cardioversion (resuscitation to sinus rhythm) while 5.1% remained in AF. The choice of energy (joules) for cardioversion (via biphasic defibrillator) was 50 in 23.7%, 100 in 27.1%, 150 in 5.1%, 200 in 30.5%, and 360 in 13.6%. The demographic and clinical characteristics of the study sample are described in Table 1.

**Table 1:** Demographic and clinical characteristics of the study participants. Values ​​are expressed as numbers and percentages unless otherwise stated. ^a^ Fisher’s exact test. ^b^ Mean value (standard deviation).

Characteristics	Ν	%
Gender		
Male	43	72.9
Female	16	27.1
Age (years)	66.3^a^	13.0^b^
Family history		
Negative family history	42	71.2
Positive family history	17	28.8
Smoking		
Non-smokers	37	62.7
Active smokers	17	28.8
Former smokers	5	8.5
Hypertension		
No	13	22.0
Yes	46	78.0
Diabetes mellitus		
No	36	61.0
Yes	23	39.0
Dyslipidemia		
No	33	55.9
Yes	26	44.1
Thyroid disease		
No	51	86.4
Yes	8	13.6
Coronary artery disease (CAD)		
No	46	78.0
Yes	13	22.0
Chronic kidney disease (CKD)		
No	56	94.9
Yes	3	5.1
Valvular heart disease		
No	54	91.5
Yes	5	8.5
Atrial fibrillation (AF)		
First diagnosed	15	25.4
Persistent AF	40	67.8
Long-term persistent AF	4	6.8
The choice of energy (joules) for synchronized direct current cardioversion (DCCV)	156.4^a^	99.6^b^
50.0	14	23.7
100.0	16	27.1
150.0	3	5.1
200.0	18	30.5
360.0	8	13.6
Outcome		
Successful AF retraction (return to sinus rhythm)	56	94.9
Unsuccessful AF retraction (remained in AF)	3	5.1

Patients underwent synchronized DCCV and cTnI was measured (by blood sampling) before DCCV and after one hour, three hours, and six hours of DCCV. Subsequent measurements showed that cTnI remained unchanged (<0.1 ng/mL) at all time points measured. The values of cTnI before and after cardioversion are presented in Table 2.

**Table 2:** Cardiac troponin I values before and after direct current cardioversion. DCCV: direct current cardioversion; cTnI: cardiac troponin I.

Measurement time points	cTnI value
Pre-DCCV	<0.1 ng/mL
1 hour post-DCCV	<0.1 ng/mL
3 hours post-DCCV	<0.1 ng/mL
6 hours post-DCCV	<0.1 ng/mL

**Figure 1:**
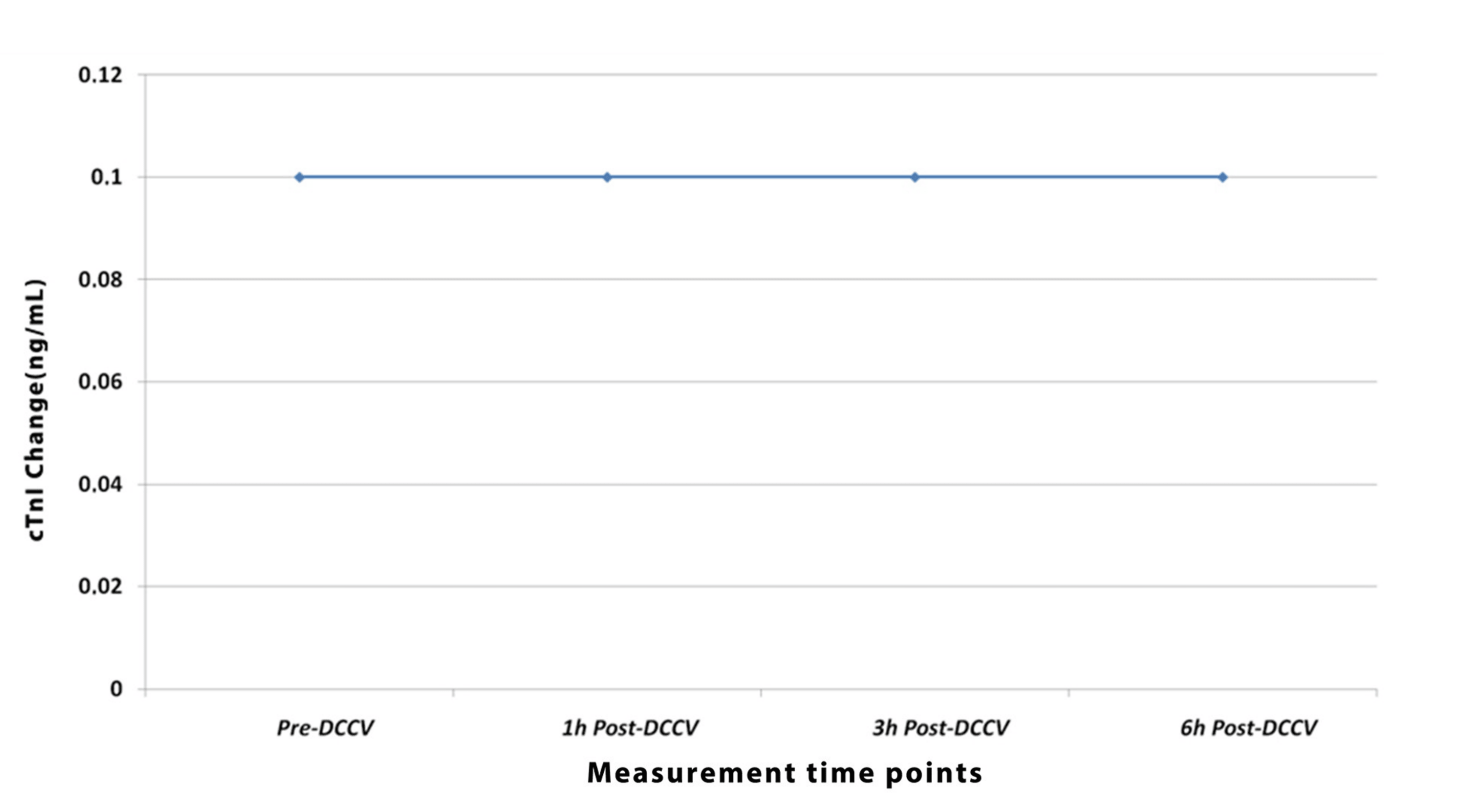
Cardiac troponin I levels pre-DCCV and post-DCCV (one, three, and six hours). DCCV: direct current cardioversion; cTnI: cardiac troponin I.

The pre-DCCV value of cTnI, as well as one hour, three hours, and six hours post-DCCV, remained unchanged. The change in cTnI after DCCV is shown in Figure 1.

**Figure 2:**
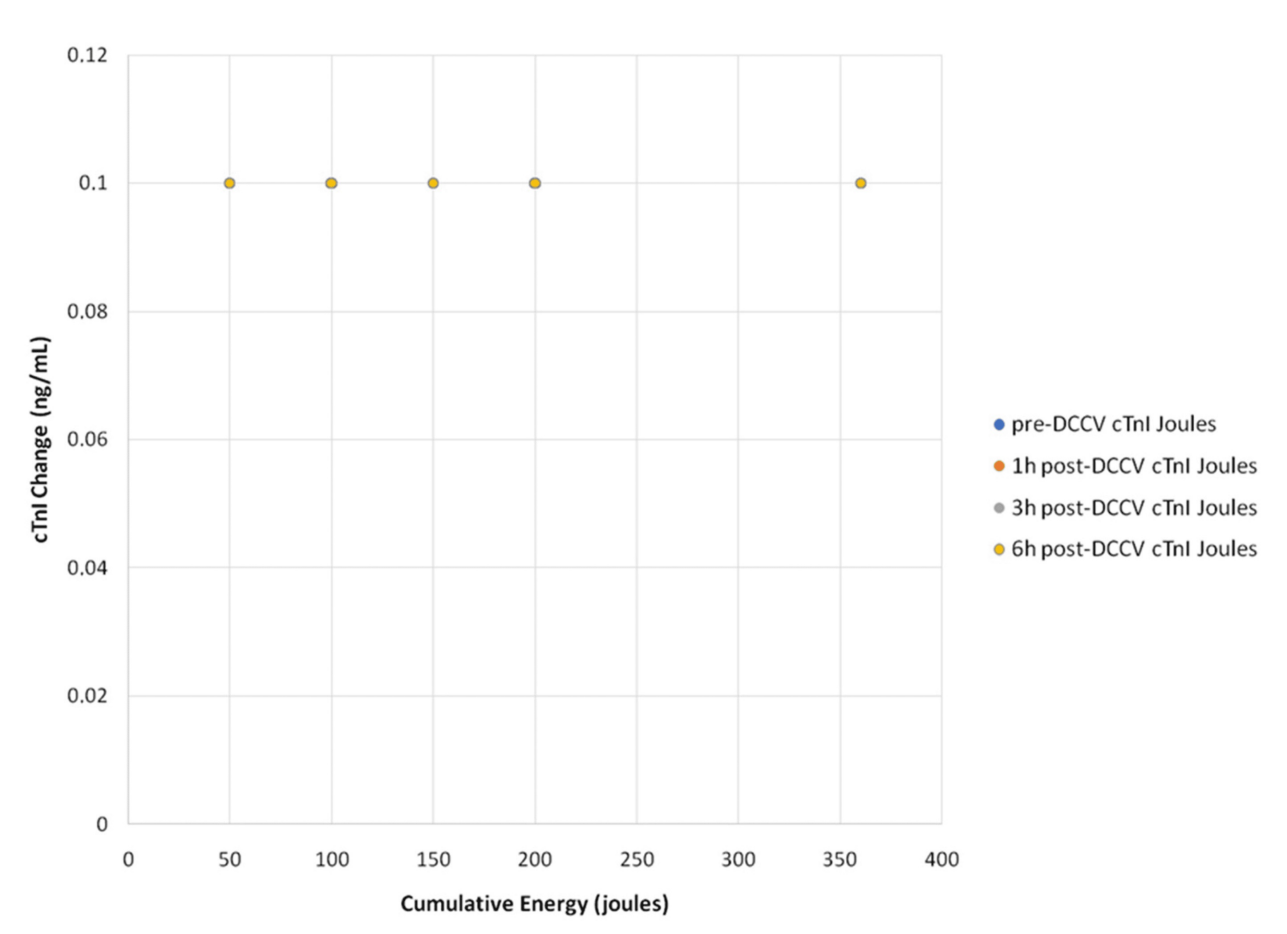
Scatter plot. The changes in the values of cardiac troponin I (cTnI) of pre-cardioversion and post-cardioversion (one, three, and six hours) are plotted against energy delivered. DCCV: direct current cardioversion.

The value of troponin I before and after one, three, and six hours of DCCV was not affected by the gradual increase of the energy (joules) used, but remained constant at cTnI < 0.1 ng/mL. The relationship between cTnI and energy (joules) is shown in Figure 2.

Correlations

Bivariate analysis revealed a statistically significant association at the 0.05 level (p < 0.05) between outcome (successful or unsuccessful AF reversion) and the choice of energy for current delivery using a biphasic manual defibrillator (p = 0.002). No statistically significant differences in outcome (successful or unsuccessful AF reversion) were found in relation to gender, age, and positive family history (p = 0.380, p = 0.963, and p = 0.197, respectively). In addition, no statistically significant differences were found in arrhythmia outcome and type of AF (p = 0.641). Table 3 shows the bivariate associations between demographic and clinical characteristics of the study patients and outcomes (successful or unsuccessful AF reversion).

**Table 3:** Bivariate correlations between demographic and clinical characteristics of the study patients and outcomes (successful or unsuccessful AF reversal) Values ​​are expressed as numbers and percentages unless otherwise stated. ^a^ Fisher’s exact test. ^b^ Mean value (standard deviation). ^c^ T-test. ^d^ Mann-Whitney test. DCCV: direct current cardioversion; AF: atrial fibrillation.

Characteristics	Outcome		p-value
	Successful AF reversion (return to sinus rhythm)	Unsuccessful AF reversion (remained in AF)	
Gender			0.380^a^
Male	40 (71.4)	3 (100.0)	
Female	16 (28.6)	0 (0.0)	
Age (years)	66.4 (13.3)	66.0 (5.2)	0.963^c^
Family history			0.197^a^
Negative family history	41 (73.2)	1 (33.3)	
Positive family history	15 (26.8)	2 (66.7)	
Smoking			0.999^d^
Non-smokers	35 (62.5)	2 (66.7)	
Current smokers	16 (28.6)	1 (33.3)	
Former smokers	5 (8.9)	0 (0.0)	
Hypertension			0.533^a^
No	12 (21.4)	1 (33.3)	
Yes	44 (78.6)	2 (66.7)	
Diabetes mellitus			0.335^a^
No	35 (62.5)	1 (33.3)	
Yes	21 (37.5)	2 (66.7)	
Dyslipidemia			0.590^a^
No	31 (55.4)	2 (66.7)	
Yes	25 (44.6)	1 (33.3)	
Thyroid disease			0.641^a^
No	48 (85.7)	3 (100.0)	
Yes	8 (14.3)	0 (0.0)	
Atrial fibrillation (AF)			0.641^d^
First diagnosed	15 (26.8)	0 (0.0)	
Persistent AF	37 (66.1)	3 (100.0)	
Long-term persistent AF	4 (7.1)	0 (0.0)	
Coronary artery disease (CAD)			0.533^a^
No	44 (78.6)	2 (66.7)	
Yes	12 (21.4)	1 (33.3)	
Chronic kidney disease (CKD)			0.853^a^
No	53 (94.6)	3 (100.0)	
Yes	3 (5.4)	0 (0.0)	
Valvular heart disease			0.763^a^
No	51 (91.1)	3 (100.0)	
Yes	5 (8.9)	0 (0.0)	
The choice of energy (joules) for synchronized DCCV^b^	145.5 (89.9)	360.0 (0.0)	0.002^d^

## Discussion

In the present study, no change was observed in cTnI level (<0.1 ng/mL) measured before DCCV and after one, three, and six hours of DCCV, but there was a correlation between arrhythmia reversion and energy (joules) used with a statistically significant difference. Furthermore, the value of cTnI was not affected (remained constant) in relation to the joules used by a biphasic external defibrillator at all time points measured (before DCCV and after one, three, and six hours of DCCV).

In a previous study conducted at the Mayo Clinic in Rochester from July 2019 to July 2020, 98 patients with persistent AF underwent cardioversion. High-sensitivity cardiac troponin I (hs-cTnI) and high-sensitivity cardiac troponin T (hs-cTnT) were measured before and at least six hours after cardioversion, and no significant increase in hs-cTnI was observed. The above study concluded that patients who show a significant increase in cardiac troponin levels after DCCV should be examined for other potential causes of myocardial injury rather than being concluded to have suffered myocardial injury as a result of DCCV [19].

Furthermore, in a study by Sless et al. [20], of 73 patients who underwent synchronized DCCV of atrial arrhythmias with and without cardiomyopathy and received troponin I before and six hours after cardioversion, no significant change in cTnI levels was observed. In other words, synchronized DCCV of an atrial arrhythmia was found not to cause myocardial injury six hours later. In another study of 48 patients with persistent AF who underwent electrical cardioversion with biphasic or single-phase defibrillators and had cTnI measured before and after six and 24 hours of cardioversion, no significant increase in cTnI was observed after cardioversion with biphasic defibrillation DCCV of atrial arrhythmias with and without cardiomyopathy and received troponin I before and six hours after cardioversion, and no significant change in cTnI levels was observed [21].

The study by Cemin et al. [22] involving 193 patients aimed to show whether myocardial damage is induced in patients with AF who undergo external ECV with normal and reduced ejection fraction. cTnI was measured 18-20 hours after cardioversion and it was found that there was no myocardial damage even in patients with a low ejection fraction. The study by Lobo et al. [23], who performed 120 cardioversions in patients with AF or AFL using biphasic defibrillators and measured hs-cTnT within six hours of cardioversion, showed that external transthoracic DCCV did not result in myocardial damage.

The use of a biphasic defibrillator for cardioversion of arrhythmia compared with a monophasic defibrillator offers better results in terms of resuscitation success because less cumulative energy is delivered and fewer shocks to the patient are required [24]. This results in no myocardial damage, which was demonstrated in the study as no change in cTnI values was observed after cardioversion with a biphasic defibrillator.

In addition, the study by Kosior et al. [25] compared the effects of single-phase versus biphasic defibrillation on myocardial injury in 63 patients with persistent AF undergoing cardioversion, measuring cTnI and myoglobin before and after six and 24 hours of cardioversion. It was shown that biphasic defibrillation did not induce myocardial damage compared to single-phase defibrillation because of the lower cumulative energy delivered. The above finding is consistent with the present study in that patients underwent biphasic defibrillation and no myocardial damage was induced.

A retrospective study by Son et al. [26] presented important findings regarding the efficacy and safety of DCCV. The study included 1718 patients mainly with persistent AF, who underwent planned DCCV, and biphasic shocks were delivered sequentially until successful cardioversion was achieved (70, 100, 150, 200, and 250 joules). In this study, the success rates were 88.6% and the average energy delivered was 144 joules. These conclusions are consistent with the findings of the present study regarding a gradual increase in energy delivered, the average energy delivered (145.6 joules), and the rates of successful cardioversion (94.9%).

The results of the present study are important and consistent with other previously reported studies, as described above. cTnI remained unchanged at all measurement times in synchronized AF using a biphasic defibrillator. Nevertheless, it is important to note that there was a correlation between converting an arrhythmia to normal sinus rhythm and the selected energy. This suggests that further studies are needed to obtain more precise results. To verify the conclusions of this study, a prospective cohort design with a larger sample size and longer follow-up is needed in the future to avoid missing subsequent changes.

Strengths and limitations

A strength of the study is that two of the researchers work in the cardiology clinic and are therefore familiar with all the procedures and protocols of the clinic. The researchers followed the instructions of the multidisciplinary team as well as the clinic's protocol. These two researchers were responsible for conducting the study, so the same criteria were strictly followed for all participants.

The present study has also several limitations. First, the sample of patients who underwent synchronized DCCV was small. In addition, the data were collected from a single hospital and thus subject to the limitations of a single-center study that follows a specific procedure not only during electrical cardioversion but also during anesthesia. However, due to technical problems with blood samples taken after DCCV (one, three, and six hours), there were some delays in sample processing time. Another limitation is that the cTnI levels were evaluated in a short period of time and some changes may not have been detected.

## Conclusions

Synchronized DCCV with a biphasic defibrillator did not affect cTnI levels in the study population, nor did it change cTnI in relation to the energy (joules) used. Therefore, DCCV with an external biphasic defibrillator does not cause myocardial damage in patients with AF.
